# Is Artificial Intelligence the New Friend for Radiologists? A Review Article

**DOI:** 10.7759/cureus.11137

**Published:** 2020-10-24

**Authors:** Sravani Gampala, Varun Vankeshwaram, Satya Siva P Gadula

**Affiliations:** 1 Radiology, GSL Medical College, Rajahmundry, IND; 2 Medicine, Zaporozhye State Medical University, Zaporozhye, UKR; 3 Internal Medicine, GSL Medical College, Rajahmundry, IND

**Keywords:** artificial intelligence in radiology, machine learning, deep learning

## Abstract

Artificial intelligence (AI) is a path-breaking advancement for many industries, including the health care sector. The expeditious development of information technology and data processing has led to the formation of recent tools known as artificial intelligence. Radiology has been a portal for medical technological advancements, and AI will likely be no dissimilar. Radiology is the platform for many technological advances in the medical field; AI can undoubtedly impact every step of a radiologist's workflow. AI can simplify every activity like ordering and scheduling, protocoling and acquisition, image interpretation, reporting, communication, and billing. AI has eminent potential to augment efficiency and accuracy throughout radiology, but it also possesses inherent drawbacks and biases.

We collected studies that were published in the past five years using PubMed as our database. We chose studies that were relevant to artificial intelligence in radiology. We mainly focused on the overview of AI in radiology, components included in the functioning of AI, AI assisting in the radiologists' workflow, ethical aspects of AI, challenges, and biases that AI experiencing together with some clinical applications of AI. Of all 33 studies, we found 15 articles discussed the overview and components of AI, five articles about AI affecting radiologist’s workflow, five articles related to challenges and biases in AI, two articles discussed ethical aspects of AI, and six articles about practical implications of AI.

We found out that the application of AI could make time-dependent tasks that can be performed effortlessly, permitting radiologists more time and opportunities to engage in patient care via increased time for consultation and development in imaging and extracting useful data from those images. AI could only be an aid to radiologists but will not replace a radiologist. Radiologists who use AI to their benefit, rather than to avoid it out of fear, might supersede those radiologists who do not. Substantial research should be done regarding the practical implications of AI algorithms for residents curriculum and the benefits of AI in radiology.

## Introduction and background

The expeditious development of information technology and data processing has led to the formation of recent tools such as artificial intelligence (AI). The term “AI” includes technical studies and innovations that utilize machines to mimic, broaden, or even enhance human knowledge [1]. Currently, AI application in radiology, specifically machine learning (ML), has become a reality in clinical practice. AI has become an ambitious initiative in every facet of radiology [2]. Developments in both imaging and computers have synergistically promoted a fast ascent in the expected AI utilization in different radiological imaging tasks, such as risk assessment, identification, diagnosis, prognosis, therapy response, and risk of reoccurrence, as well as in multi-omics disease revelation [3]. Radiology varies from other image identification applications of AI algorithms. Computed tomography or magnetic resonance can comprise thousands of images as a counter to a single image, increasing the complexity of required computational algorithms [4]. Radiomics is an extension of computer-aided diagnosis that has been determined as the transformation of images to minable data. Radiomics is a compound multi-step process that helps clinical decision-making and prognosis prediction by integrating information from various tests involving clinical, molecular, imaging, and genomic data [5]. Some of the internal and external disadvantages in AI methods are impeding its implementation in the clinical area [2].

Some of the clinical applications of AI, the progression in deep learning, and the standard of AI are quickly improving for breast imaging. It will play an essential role in all steps of mammography and digital breast tomosynthesis from image creation and deionizing to risk assessment, cancer detection, and finally, therapy selection and response prediction [6]. AI has a significant role in the interpretation of breast cancer; Herent et al. demonstrated the capability of a deep learning model to discriminate between benign and malignant lesions using magnetic resonance imaging (MRI) and identify various histological subtypes of breast cancer [7]. AI has multiple implications in thoracic imaging such as lung nodule assessment, tuberculosis or pneumonia detection, or estimation of diffuse lung diseases. Currently, 14 common anomalies, when presented as isolated findings, can be detected by algorithms. Chest radiography is a perfect domain, and chest computed radiography is another large field of application for AI.

Regarding the implications in oncology, AI technologies have been incorporated into the computer-aided diagnosis technologies such as lesion detection and analysis of various parameters [8]. In radiotherapy, from the initial patient encounter to pretreatment disease outcome and toxicity prediction, AI can assist in every step. AI may subsequently aid in treatment planning, dose optimization, and support a greater level of safety, quality, and efficiency of care [9].

Despite the hype and expectations given to AI, we could not find many related articles that reported AI implications, especially for consumers and patients. Currently, the most common AI usage for patients and comers lies in the online discussion forms [10]. We find a lack of clarity about the functioning of algorithms; also, there are issues regarding the accountability of machine-made mistakes [11]. In this review, we would like to give a brief overview of AI and terminology, various subfields, and the components of AI and few clinical applications, the challenges that AI faces, biases that could take place during the implication, and ethical aspects of AI.

## Review

Methods

We followed the PRISMA guidelines and used PubMed as our main database to search for articles that are relevant to artificial intelligence and related components and ethical aspects as well as AI implications. We have searched in PubMed using AI in radiology and we found 8,281 articles further by using filters for the last five years we got 4196 articles. Moreover, we narrowed our search according to our requirements such as AI in radiology overview, AI ethics, AI clinical applications and we finally selected 34 articles that are relevant to our article. We performed a literature search by using keywords artificial intelligence radiology, machine learning, deep learning.

We included studies that are suitable for the topic and peer-reviewed. All the studies that are included were published in English within the last five years from the search date (2015-2020).

We focused on the overview of AI in radiology, components included in the functioning of AI, AI assisting in the radiologists' workflow, ethical aspects of AI, challenges, and biases that AI experiencing together with some clinical applications of AI. All the data was collected ethically and legally. Quality appraisal of the studies included was done using the New-Castle Ottawa Scale for observational studies, and AMSTAR checklists for systematic reviews/meta-analysis.

Results

By following the search criteria mentioned in the Methods section, we gathered a total of 34 articles that were relevant to Artificial Intelligence. Among them, 20 were review articles, one systematic review, three published papers, two editorials, six retrospective observational studies, two comparative studies. All the studies included were peer-reviewed and were included only after appropriate quality assessment tools.

Discussion

Few Words About AI

Artificial intelligence (AI) is a path-breaking advancement for many industries, including the health care sector. Radiology has been a portal for medical technological advancements, and AI will likely be no dissimilar [12]. AI in radiology showed its implications dated back to at least 1994 [13]. AI is defined as regulation within computer science in which an artificial system can imitate human cognitive ability by having the potential to learn and solve problems. To perform this, the artificial system should recognize its environment, execute pattern perception, and consequently plan and operate the appropriate course of action [14]. AI could be further sub-classified to narrow (or weak) and broad (or strong AI). Narrow AI mostly performs a single task, whereas broad AI can perform a variety of tasks. Narrow AI uses algorithms typically trained using supervised learning; it is referred to as augmented intelligence as it plays an assistive role that amplifies human intelligence rather than replaces it. Broad AI is also called artificial general intelligence, which refers to an entity that can learn and self-aware and execute a diversity of tasks [15]. Narrow AI is mostly pertinent to diagnostic imaging; broad AI has limited health care [16].

Overview of Some Functional Units of AI

Machine learning (ML) is a portion of AI that concerns the construction of computer algorithms that automatically improve with experience without prominent programming of the decision-making rules [17]. ML has two ways of supervised and unsupervised learning. As radiology is a data-driven discipline, in supervised learning, the computer is handed with labeled data to train the algorithms, and it is used in narrow AI. In contrast, in unsupervised learning, unlabeled data is handed to the computer that the algorithms must learn to self-classify, and it is used in broad AI [15]. Deep learning (DL) is a subset of machine learning and is involved with algorithms influenced by the brain's structure and the function called artificial neural networks (ANN) [17]. These neural networks are composed of multiple layers, from which the algorithms can draw relevant features. ANNs have become the anchor for AI amelioration for diagnostic imaging [15]. DL techniques are classified into five sections such as classification, object detection, semantic segmentation, image processing, and natural language processing [18]. The principal position of radiology in health care, together with digitalization and the emergence of Picture Archiving and Communication Systems (PACS), has galvanized to the exponential growth of stored digital imaging datasets in the last two decades. The efficiency and accessibility of the healthcare system have improved by applying AI algorithms to the data sets [1].

AI Affecting Radiology

Radiology is the platform for many technological advances in the medical field; unlike most recent radiology technologies, AI can undoubtedly impact every step of a radiologist's workflow. AI can simplify every activity like ordering and scheduling, protocoling and acquisition, image interpretation, reporting, communication, and billing [12]. AI can be used even during patient's scheduling. Curtis et al. showed an AI model that could precisely estimate wait times or appointment delays for radiography, ultrasound, CT, MRI. By communicating these times to patients can result in enhanced patient satisfaction, and it also helps to identify process improvement opportunities diligently; therefore, augment the number of tests accomplished by any resource [19]. At the time of point of care for medical decision support in requests for imaging, AI algorithms can be used to scrutinize a patient's medical records and decide the suitableness of imaging and provide advice for which imaging exam would be most appropriate [20]. Some AI systems have demonstrated assurance in de-noising data obtained at notably lower radiation levels than previously required, resulting in image acquisition optimization with dramatically low radiation exposure [21]. AI also helps minimize artifacts by applying image processing algorithms during or after reconstruction steps [22]. In-depth learning programs can facilitate automated image interpretation; Rajpurkar et al. showed a model for detecting pneumonia in chest X-ray with diagnostic accuracy like radiologists [23]. Despite this possibility, there is currently no commercially available solution to interpret images and give a report. With the advent of AI, there are many other additional opportunities like lesion measurements with volumetric calculations using image segmentation driven by deep learning. The training AI system could also extract cancer history from the endoscopic mucosal resection (EMR) and provide the radiologist with appropriate diagnosis during diagnosis, which improves workflow efficiency. The tasks that require extensive training could be automated by using AI, which provides additional time to dedicate to the new opportunities like participation in patient care. Slowly, AI is redefining the duties of radiologists [12].

Challenges and Biases in AI

AI has eminent potential to augment efficiency and accuracy throughout radiology, but it also possesses inherent drawbacks and biases. AI requires extensive data to design supreme quality training sets for algorithms to learn, which can be expensive and time-taking for radiologists to prepare [12]. AI can be problematic and widespread use of AI-dependent intelligent and autonomous equipment can increase systemic risks of harm, raise the likelihood of errors with significant effects, and increase compound ethical and societal issues [24]. A fundamental challenge in AI is the data that is used in machine learning do not be necessarily generalized. As a result, even in confined tasks like image interpretation, AI can make inaccurate diagnoses due to differences in the training and real-world populations [25]. Another challenge is that the professional knowledge algorithms' dynamic changes need to reflect up-to-date information [26]. There are some disparities in the accessibility of AI, especially for the smaller or resource poor-hospitals that may be in a deficit of the technology, skills, and resources to maintain complex AI systems [24]. The major drawback of AI is accountability, that is, when errors are happened using AI, who is accountable for the computer? Is it the radiologist, the AI application itself, or the company that developed the AI application responsible? The inferences involved in developing an algorithm should be explained in a way that can be appreciated by humans, such as bounding boxes or saliency maps. If the radiologist does not understand the process behind an algorithm's working, how can radiologists be entirely responsible for the errors? To tackle this “black box” problem, many groups, together with the American Medical Association, develop policies that insist developers furnish explainability and transparency in the algorithm development [11].

There are certain types of bias associated with AI. Automation bias is the propensity for humans to favor derived machine decisions, disregarding contrary, or conflicting human decisions. Automation bias can cause errors of omission and commission. When humans fail to observe or disregard the failure of an AI tool, omission error occurs. Despite the contrary evidence, when one erroneously implements a machine's decision, commission error occurs. In the resource-poor population, automation bias might be magnified when there is no local radiologist [24]. Algorithmic bias occurs when an algorithm compounds and amplifies the existing inequities in socioeconomic status, race, ethnic background, religion, gender, disability, or sexual disorientation during its application, which adversely impacts equity in health systems. The factors contributing to algorithm bias lack exact definitions and standards of fairness, inadequate contextual specificity, and the black-box nature of algorithms [27].

Ethical Aspects of AI

According to the summary of the joint European and North American multi-society statement, AI should esteem human rights and liberty, including privacy and dignity. To establish patient and provider trust in AI, it should have extreme transparency and dependability. However, the level of transparency is debatable because an expansive definition of transparency could jeopardize the patient's privacy by revealing personal data hidden in primary data sets. Final responsibility and accountability for AI lie with human designers and operators for the predictable future. The radiology network should begin currently to create codes of ethics and practice for AI. These codes ought to advance any utilization that helps patients benefit all and should hinder the utilization of radiology information and calculations for financial profit without those two ascribes. End ethical utilization of AI in radiology ought to advance prosperity, limit hurt, and guarantee that the advantages and damages are conveyed among the potential partners equitably. New ethical problems will show up rapidly and routinely, and our understanding of them will change over time. Thus, though it is necessary to contemplate the ethics of AI in radiology now, it also will be essential to evaluate the subject continuously as our perception of its impact and ability increases and to return to the AI tools getting used in radiology to assess whether they meet the updated rules and standards. Radiologists will remain eventually answerable for quiet consideration and should obtain new abilities to put forth a valiant effort for patients in the new AI environment [24].

Practical Applications of AI

There is currently an absence of direction and proof of how AI would benefit patients and consumers. Also, there is little experience using AI for patient well-being. Perhaps rather than specializing in information and algorithms, analyzers should interact with patients and consumers early in the AI research agenda to confirm we ask the pertinent questions. That critical use cases and demanding contexts are known together with patients and consumers. Without a transparent understanding of why patients and consumers need AI within the first place, how AI could support people with their healthcare needs, and what are the capabilities and limitations of AI, it is tough to imagine the varieties of AI applications that would have a significant and sustainable impact on individuals' daily lives [9]. Table 1 shows some of the practical implications and efficiency of AI.

**Table 1:** Table showing practical implications of AI AI: Artificial intelligence; CT: Computed tomography; MRI: Magnetic resonance imaging; MR: Magnetic resonance; CNN: Convolutional neural network.

Author	Study	Conclusion
Hamm et al. [28]	Artificial intelligence and radiomics in MRI-based prostate diagnostics	This study concludes that AI targets on the identification and classification of prostate cancer, but also attempts to classify aggressive tumor nature according to the Gleason score.
Jermyn et al. [29]	Neural networks improve brain cancer detection with Raman spectroscopy in the presence of operating room light artifacts	This study states that by providing molecular information that distinguishes between a normal brain and cancer tissue to Raman spectroscopy, it can detect invasive brain cancer in glioma patients.
Charron et al. [30]	Automatic detection and segmentation of brain metastases on multimodal MR images with a deep convolutional neural network	This study demonstrated that a deep network approach is propitious for the spotting and the segmentation of brain metastases on multimodal MRI.
Rodriguez-Ruiz et al. [31]	Stand-alone Artificial Intelligence for breast cancer detection in mammography: comparison with 101 radiologists	This study concluded that the AI system's performance was statistically non-inferior to that of the 101 radiologists; it achieved a cancer detection accuracy comparable to an average breast radiologist in this retrospective setting
Ehteshami Bejnordi et al. [32]	Diagnostic assessment of deep learning algorithms for detection of lymph node metastases in women with breast cancer	This study demonstrated that some deep learning algorithms achieved better diagnostic performance than a panel of 11 pathologists participating in a simulation exercise designed to mimic routine pathology workflow. The algorithm performance was comparable with an expert pathologist interpreting whole-slide images without time constraints.
Urakawa et al. [33]	Detecting intertrochanteric hip fractures with orthopedist-level accuracy using a deep convolutional neural network	This study concluded that the convolutional neural networks’ (CNN) performance exceeded that of orthopedic surgeons in identifying intertrochanteric hip fractures from proximal femoral radiographs under limited conditions. CNN has a considerable potential to be a useful tool for screening for fractures on plain radiographs, especially in the emergency room, where orthopedic surgeons are not readily available.
Liu et al. [34]	Clinical application of artificial intelligence recognition technology in the diagnosis of stage T1 lung cancer	This study states that the automatic learning of early lung cancer, chest CT images by artificial intelligence can achieve high sensitivity and specificity of early lung cancer detection and could assist doctors in the diagnosis of lung cancer.

Limitations

Some limitations of this review article were that the data was collected from the last five years. We have precluded the papers that are published in other languages. There is vast data published about AI, and it is difficult to analyze every concept; hence, most essential topics are discussed.

## Conclusions

We conclude that radiologists are in a distinctive position to encourage the AI revolution in healthcare by their direct relationship with a remarkable amount of data. The number of radiological tests conducted each year has increased rapidly over the last two years, almost increasing two-fold every 10 years. The application of AI can make time-dependent tasks that can be performed effortlessly, permitting radiologists more time and opportunities to engage in patient care via increased time for consultation and development in imaging and extracting useful data from those images. AI could only be an aid to radiologists but will not replace a radiologist. Radiologists who use AI to their benefit, rather than to avoid it out of fear, might supersede those radiologists who do not. There is a smaller number of articles reporting AI applications explicitly designed for patients or consumers. Extensive research needs to be done on the improvement of ethical aspects and transparency regarding decision-making or potential errors associated with AI-powered technologies.

## References

[1] SFR-IA Group; CERF; French Radiology Community (2018). Artificial intelligence and medical imaging 2018: French Radiology Community white paper. Diagn Interv Imaging.

[2] Martín Noguerol T, Paulino-Godino F, Martín-Valdivia MT, Menias CO, Luna A (2019). Strengths, weaknesses, opportunities, and threats analysis of artificial intelligence and machine learning applications in radiology. J Am Coll Radiol.

[3] Giger ML (2018). Machine learning in medical imaging. J Am Coll Radiol.

[4] McBee MP, Awan OA, Colucci AT (2018). Deep learning in radiology. Acad Radiol.

[5] Rizzo S, Botta F, Raimondi S, Origgi D, Fanciullo C, Morganti AG, Bellomi M (2018). Radiomics: the facts and the challenges of image analysis. Eur Radiol Exp.

[6] Geras KJ, Mann RM, Moy L (2019). Artificial intelligence for mammography and digital breast tomosynthesis: current concepts and future perspectives. Radiology.

[7] Herent P, Schmauch B, Jehanno P (2019). Detection and characterization of MRI breast lesions using deep learning. Diagn Interv Imaging.

[8] Setzer FC, Shi KJ, Zhang Z, Yan H, Yoon H, Mupparapu M, Li J (2020). Artificial intelligence for the computer-aided detection of periapical lesions in cone-beam computed tomographic images. J Endod.

[9] Deig CR, Kanwar A, Thompson RF (2019). Artificial intelligence in radiation oncology. Hematol Oncol Clin North Am.

[10] Lau AYS, Staccini P (2019). Artificial intelligence in health: new opportunities, challenges, and practical implications. Yearb Med Inform.

[11] Crigger E, Khoury C (2019). Making policy on augmented intelligence in health care. AMA J Ethics.

[12] Syed AB, Zoga AC (2018). Artificial intelligence in radiology: current technology and future directions. Semin Musculoskelet Radiol.

[13] Kahn CE Jr (1994). Artificial intelligence in radiology: decision support systems. Radiographics.

[14] Hosny A, Parmar C, Quackenbush J, Schwartz LH, Aerts HJWL (2018). Artificial intelligence in radiology. Nat Rev Cancer.

[15] Sogani J, Allen B Jr, Dreyer K, McGinty G (2020). Artificial intelligence in radiology: the ecosystem essential to improving patient care. Clin Imaging.

[16] Rezazade Mehrizi MH, van Ooijen P, Homan M (2020). Applications of artificial intelligence (AI) in diagnostic radiology: a technography study (Online ahead of print). Eur Radiol.

[17] Wong SH, Al-Hasani H, Alam Z, Alam A (2019). Artificial intelligence in radiology: how will we be affected?. Eur Radiol.

[18] Ueda D, Shimazaki A, Miki Y (2019). Technical and clinical overview of deep learning in radiology. Jpn J Radiol.

[19] Curtis C, Liu C, Bollerman TJ, Pianykh OS (2018). Machine learning for predicting patient wait times and appointment delays. J Am Coll Radiol.

[20] Lu W, Onofrey JA, Lu Y, Shi L, Ma T, Liu Y, Liu C (2019). An investigation of quantitative accuracy for deep learning based denoising in oncological PET. Phys Med Biol.

[21] Sachs PB, Gassert G, Cain M, Rubinstein D, Davey M, Decoteau D (2013). Imaging study protocol selection in the electronic medical record. J Am Coll Radiol.

[22] Zhu B, Liu JZ, Cauley SF, Rosen BR, Rosen MS (2018). Image reconstruction by domain-transform manifold learning. Nature.

[23] Rajpurkar P, Irvin J, Ball RL (2018). Deep learning for chest radiograph diagnosis: a retrospective comparison of the CheXNeXt algorithm to practicing radiologists. PLoS Med.

[24] Geis JR, Brady AP, Wu CC (2019). Ethics of artificial intelligence in radiology: summary of the joint European and North American multisociety statement. Can Assoc Radiol J.

[25] Challen R, Denny J, Pitt M, Gompels L, Edwards T, Tsaneva-Atanasova K (2019). Artificial intelligence, bias and clinical safety. BMJ Qual Saf.

[26] Magrabi F, Ammenwerth E, McNair JB (2019). Artificial intelligence in clinical decision support: challenges for evaluating AI and practical implications. Yearb Med Inform.

[27] Panch T, Mattie H, Atun R (2019). Artificial intelligence and algorithmic bias: implications for health systems. J Glob Health.

[28] Hamm CA, Beetz NL, Savic LJ, Penzkofer T (2020). Artificial intelligence and radiomics in MRI-based prostate diagnostics (Article in German). Radiologe.

[29] Jermyn M, Desroches J, Mercier J (2016). Neural networks improve brain cancer detection with Raman spectroscopy in the presence of operating room light artifacts. J Biomed Opt.

[30] Charron O, Lallement A, Jarnet D, Noblet V, Clavier JB, Meyer P (2018). Automatic detection and segmentation of brain metastases on multimodal MR images with a deep convolutional neural network. Comput Biol Med.

[31] Rodriguez-Ruiz A, Lång K, Gubern-Merida A (2019). Stand-alone artificial intelligence for breast cancer detection in mammography: comparison with 101 radiologists. J Natl Cancer Inst.

[32] Ehteshami Bejnordi B, Veta M, Johannes van Diest P (2017). Diagnostic assessment of deep learning algorithms for detection of lymph node metastases in women with breast cancer. JAMA.

[33] Urakawa T, Tanaka Y, Goto S, Matsuzawa H, Watanabe K, Endo N (2019). Detecting intertrochanteric hip fractures with orthopedist-level accuracy using a deep convolutional neural network. Skeletal Radiol.

[34] Liu X, Zhou H, Hu Z, Jin Q, Wang J, Ye B (2019). Clinical application of artificial intelligence recognition technology in the diagnosis of stage T1 lung cancer (Article in Chinese). Zhongguo Fei Ai Za Zhi.

